# Enzymatic saccharification of shrub willow genotypes with differing biomass composition for biofuel production

**DOI:** 10.3389/fpls.2013.00057

**Published:** 2013-03-25

**Authors:** Michelle J. Serapiglia, Michele C. Humiston, Haowen Xu, David A. Hogsett, Ramón M. de Orduña, Arthur J. Stipanovic, Lawrence B. Smart

**Affiliations:** ^1^Department of Horticulture, New York State Agricultural Experiment Station, Cornell UniversityGeneva, NY, USA; ^2^Department of Food Science, New York State Agricultural Experiment Station, Cornell UniversityGeneva, NY, USA; ^3^Mascoma Corporation, LebanonNH, USA; ^4^Department of Chemistry, State University of New York College of Environmental Science and ForestrySyracuse, NY, USA

**Keywords:** bioenergy, biomass, ethanol, hydrolysis, *Salix*

## Abstract

In the conversion of woody biomass feedstocks into liquid fuel ethanol, the pretreatment process is the most critical and costly step. Variations in biomass composition based on genetic differences or environmental effects have a significant impact on the degree of accessibility accomplished by pretreatment and subsequent sugar release by enzymatic hydrolysis. To evaluate this, biomass from 10 genetically diverse, genotypes of shrub willow (*Salix* spp.) was pretreated with a hot-water process at two levels of severity, hydrolyzed using a combination of two commercial enzyme cocktails, and the release of hexose and pentose monomers was quantified by high-performance liquid chromatography. Among the genotypes selected for analysis, cellulose content ranged from 39 to 45% (w/w) and lignin content ranged from 20 to 23% (w/w) at harvest. Differences in the effectiveness of the pretreatment process were observed among the various willow genotypes. Correlations were identified between total sugar release and % cellulose and % lignin content. There was a significant effect of pretreatment severity on polysaccharide accessibility, but the response to pretreatments was different among the genotypes. At the high severity pretreatment ‘SV1’ was the least recalcitrant with sugar release representing as much as 60% of total biomass. These results suggest that structural, as well as chemical characteristics of the biomass may influence pretreatment and hydrolytic efficiency.

## INTRODUCTION

The reduction of US dependency on foreign oil will require the production of domestic renewable transportation fuels from lignocellulosic biomass as alternative energy sources. The Energy Independence Security Act of 2007 has mandated an increase in the renewable fuel standard to 36 billion gallons of renewable fuels by 2022 ( H.R., 2007). To reach this goal within the next decade and prevent competition for food crops such as corn, the conversion processes of lignocellulosic biomass into fermentation products, such as ethanol or butanol has to be improved. Dedicated energy crops, including perennial grasses and short-rotation woody crops, such as hybrid poplar (*Populus* spp.) and shrub willow (*Salix* spp.), will be crucial in the economic viability of renewable fuel production.

The feasibility of generating biofuels from lignocellulosic energy crops is largely dependent on cost reductions throughout the production cycle and conversion process. Converting lignocellulosic material into ethanol involves four major steps: pretreatment, hydrolysis, fermentation, and product purification or distillation ( Lynd, 1996). Pretreatment processes break apart the highly recalcitrant lignocellulosic material mechanically or chemically to make cellulose and hemicelluloses more accessible for hydrolysis. This requires a large amount of energy and is the most expensive step both economically and energetically ( Lynd, 1996; Himmel et al., 2007; Yang and Wyman, 2008). Overcoming this recalcitrance and improving cellulose digestibility are key research areas for making cellulosic ethanol profitable and involve improvements in pretreatments and hydrolysis. However, there is also potential for cost reductions by identifying and breeding feedstocks with improved sugar release capabilities that are optimal for biofuel production ( Guo et al., 2009; Brereton et al., 2010).

Shrub willow bioenergy crops have many desirable characteristics for feedstock and biomass production. With their coppicing ability and vigorous juvenile growth they can produce high biomass yield (>11 odt (oven dried tonnes) ha^-1^ year^-1^) on marginal land not suitable for conventional food crops ( Volk et al., 2011). Since there has been little cultivation and domestication of shrub willow crops, there is a broad genetic resource available for breeding and a high level of genetic diversity to utilize in the genus *Salix*, consisting of over 300 species. Selection for pest and disease resistance and improvements in yield has been successful in Sweden, UK, and US ( Ahman and Larsson, 1994; Karp et al., 2011; Höglund et al., 2012; Serapiglia et al., 2012).

It has been shown that shrub willow has high phenotypic variation for biomass composition ( Serapiglia et al., 2009), owing to differences in cell wall characteristics. Several studies have shown a relationship between lower lignin content in the biomass and improved sugar release, indicating that lignin contributes to the recalcitrance of the cell wall ( Chen and Dixon, 2007; Jackson et al., 2008; Studer et al., 2011). However, lignin content is not the only contributing factor for sugar release. Further research is required to elucidate the impact of cell wall chemistry and its components on polysaccharide accessibility to improve breeding strategies.

In this study, we investigated the biomass compositional variation among 30 unique genotypes of shrub willow planted at a single site and examined sugar release following enzymatic hydrolysis of untreated biomass. Further examination of 10 selected genotypes included a hot-water pretreatment at two severity levels followed by enzymatic saccharification to quantify sugar release and the conversion to ethanol utilizing a simultaneous saccharification and fermentation (SSF) method. The goal of this project was to determine if differences in biomass compositional characteristics among genotypes of shrub willow affect the release of sugars from enzymatic saccharification and the conversion efficiency to ethanol.

## MATERIALS AND METHODS

### SOURCE MATERIAL AND BIOMASS COLLECTION

Thirty genotypes of willow were hand planted in May 2006 using 25-cm cuttings in double row spacing in four completely randomized blocks in the 2006 Yield Trial in Constableville, NY, USA (**Table 1**). The trial was coppiced at the end of the first growing season, and after the third post-coppice season, willow stems were harvested in December 2009. Stems were chipped and the four replicates pooled. Biomass yield was measured and a sub-sample was collected, dried, and weighed to determined moisture content at harvest, which allowed estimation of dry weights. For this, chips were dried to a constant weight at 60°C and ground in a Wiley mill through a 20-mesh screen. Further fine milling to 0.5 mm particle size was performed using an MF 10 analytical mill (IKA, Wilmington, NC, USA). In addition, a 25-cm section was collected from the middle of a typical canopy stem of one plant in each plot to determine wood density by volumetric displacement (TAPPI Standard T 258 om-06, 2006).

**Table 1:** Shrub willow genotypes used in this study harvested from the 2006 Constableville yield trial.

Clone ID/ cultivar epithet	Species/pedigree	Source
‘SV1’	*Salix* × *dasyclados*	University of Toronto
‘SX61’	*S. sachalinensis*	University of Toronto
‘SX64’	*S. miyabeana*	University of Toronto
‘S25’	*S. eriocephala*	University of Toronto
94001	*S. purpurea*	Blossvale, NY
9832-49	*S. eriocephala*	Bred in 1998
9837-77	*S. eriocephala*	Bred in 1998
00X-026-082	*S. eriocephala*	Bred in 2000
00X-032-094	*S. eriocephala*	Bred in 2000
‘Fish Creek’	*S. purpurea*	Bred in 1998
‘Wolcott’	*S. purpurea*	Bred in 1998
‘Onondaga’	*S. koriyanagi* × *S. purpurea*	Bred in 1999
‘Allegany’	*S. koriyanagi* × *S. purpurea*	Bred in 1999
‘Oneonta’	*S. purpurea* × *S. miyabeana*	Bred in 1998
‘Oneida’	*S. purpurea* × *S. miyabeana*	Bred in 1999
‘Millbrook’	*S. purpurea* × *S. miyabeana*	Bred in 1999
‘Saratoga’	*S. purpurea* × *S. miyabeana*	Bred in 1999
‘Sherburne’	*S. sachalinensis* × *S. miyabeana*	Bred in 1998
‘Canastota’	*S. sachalinensis* × *S. miyabeana*	Bred in 1999
‘Cicero’	*S. sachalinensis* × *S. miyabeana*	Bred in 1998
‘Marcy’	*S. sachalinensis* × *S. miyabeana*	Bred in 1998
‘Preble’	*S. viminalis* × (*S. sachalinensis* × *S. miyabeana*)	Bred in 2001
‘Otisco’	*S. viminalis* × *S. miyabeana*	Bred in 1999
‘Verona’	*S. viminalis* × *S. miyabeana*	Bred in 1999
‘Tully Champion’	*S. viminalis* × *S. miyabeana*	Bred in 1999
‘Fabius’	*S. viminalis* × *S. miyabeana*	Bred in 1999
‘Taberg’	*S. viminalis* × *S. miyabeana*	Bred in 1999
‘Erie’	*S. viminalis* × *S. miyabeana*	Bred in 1999
‘Truxton’	*S. viminalis* × *S. miyabeana*	Bred in 1999
‘Owasco’	*S. viminalis* × *S. miyabeana*	Bred in 1999

### HIGH-RESOLUTION THERMOGRAVIMETRIC ANALYSIS

Unextracted willow biomass samples were analyzed by thermogravimetric analysis (Thermogravimetric Analyzer 2950, TA Instruments, New Castle, DE, USA with TA Universal Analysis 2000 software) according to Serapiglia et al. (2009). Each biomass sample was analyzed with three instrumental replicates. Results obtained from this analysis were expressed as % cellulose, hemicelluloses, lignin, and ash as a proportion of total dry biomass.

### HOT-WATER PRETREATMENT

Dried, unextracted, ground biomass was soaked to saturation in water overnight at 4°C. All samples were filtered through Büchner funnels with 15 cm Whatman filters (grade 1). Dry weight was determined in triplicate by drying sub-samples of approximately 2 g overnight at 105°C. The willow biomass at a solid loading of 20% (w/w) with water was pretreated in 316 stainless steel tube reactors fitted with 316 stainless steel caps (Swagelok, USA) to prevent evaporation of the liquid fraction. All samples were pretreated at 200°C in a fluidized sand bath (Techne Precision, USA) with two different resonance times of 5 and 9 min, allowing for 5 min of heat-up time. All samples were pretreated in duplicate. Rapid cooling was achieved by quenching all reactors in an ice bath for 10 min. All samples were transferred to storage containers. Moisture content and total solids was determined for all samples before continuing with enzymatic hydrolysis or ethanol production. The pretreatment was adjusted to a log *R*_0_ severity of 3.6 and 3.9 (Eq. 1). Severity was defined as

(1) $$R_{0}=t\,\cdot e^{\frac{T-100}{14.75}},$$

where *t* is the time (minute) and *T* the temperature (°C).

### ENZYMATIC HYDROLYSIS

Enzymatic hydrolysis was performed with non-pretreated, non-extracted biomass from all 30 willow genotypes and with pretreated biomass from 10 selected genotypes according to an established National Renewable Energy Laboratory (NREL) protocol ( Selig et al., 2008). For the non-pretreated biomass samples, 200 mg of dry biomass was added to 20 mL scintillation vials. For the pretreated biomass, wet biomass equivalent to 200 mg dry biomass was used. Five milliliters of a 0.1-M sodium citrate buffer (pH 5) and 500 μL of a 100-μg mL^-1^ natamycin solution were added to all samples. Two commercially produced enzyme mixes were used for hydrolysis: 100 μL of Cellic^®^ CTec2 (Novozymes, Wilmington, DE, USA), which is a blend of cellulases, β-glucosidases, and hemicellulase and 20 μL of Cellic^®^ HTec2 (Novozymes), which contains additional endoxylanases. All samples were brought to 10 mL total volume using deionized water. The samples were capped and placed in a shaker-water bath at 50°C for 48 h at 200 rpm. Following incubation, samples were filter sterilized (0.45 μm nylon, Grace, Deerfield, IL, USA) for sugar analysis. The collected dry biomass was weighed, and sub-samples were collected for moisture and total solid determination.

### SUGAR QUANTIFICATION

Sugars were quantified by high-performance liquid chromatography (HPLC) using a Shimadzu Prominence System (Columbia, MD, USA) consisting of a DG-20A3 in-line degasser, LC-20AB binary pump, SIL-10AD auto-sampler, CTO-20AC column oven, and RID-10A refractive index detector. Following filtration (0.22 μm, nylon membrane, Whatman) 20 μL sample was injected and separated using a sulfonated styrene-divinylbenzene stationary phase (300 mm × 7.8 mm i.d., Aminex HPX-87P, Bio-Rad, Hercules, CA, USA) preceded by a cartridge type pre-column (30 mm × 4.6 mm i.d., de-ashing phase, Bio-Rad) at 85°C. The mobile phase consisted of ASTM Class I water (Arium 611UV, Sartorius, Germany) and the flow rate was 0.5 mL min^-1^. Data analysis was carried out using the software supplied (LC Solution v.1.23, Shimadzu).

### ETHANOL PRODUCTION BY SSF

Simultaneous saccharification and fermentation was performed on pretreated biomass from 10 selected genotypes. Fermentation studies were performed at a 20-mL scale in sealed serum bottles using unextracted, non-dried pretreated material at a final concentration of 50 g L^-1^ (oven dried basis). A medium comprised of 12 g L^-1^ corn steep liquor and 0.5 g L^-1^ diammonium phosphate, buffered by 50 mM acetate and adjusted to an initial pH of 5.5, was used. Penicillin G was added at a final concentration of 30 μg mL^-1^ to inhibit potential bacterial contaminants. Hydrolysis was accomplished through the addition of 60 μL Cellic^®^ CTec2 and 6 μL Cellic^®^ HTec2. An engineered, xylose-fermenting strain of *Saccharomyces cerevisiae* (Mascoma Corporation) was inoculated at 0.5 g L^-1^ dry cell weight (DCW) for fermentation at 35°C at 150 rpm. After 73 and 120 h of fermentation, samples were withdrawn, filtered, acidified, and analyzed by HPLC for ethanol, organic acids, and sugar monomers using an Agilent 1100 System with a refractive index detector (Santa Clara, CA, USA). All samples were separated on an Aminex HPX-87H column with a mobile phase consisting of 0.01 N sulfuric acid with a flow rate of 0.6 mL min^-1^ ( Sluiter et al., 2008). All samples were analyzed using ChemStation (Agilent).

### STATISTICAL ANALYSIS

All statistical analyses were performed using SAS^®^ version 9.2 at a critical α = 0.05 ( SAS Institute Inc., 2000–2004). SAS PROC UNIVARIATE was used to summarize the data distribution for all variables analyzed. PROC GLM was used to perform analysis of variance. When a significant difference (*P* < 0.05) was observed, Tukey’s mean studentized range test was used for pairwise comparisons. PROC CORR was used to identify any significant correlations among variables obtained in this study.

## RESULTS

### BIOMASS COMPOSITION AND WOOD DENSITY

**FIGURE 1:**
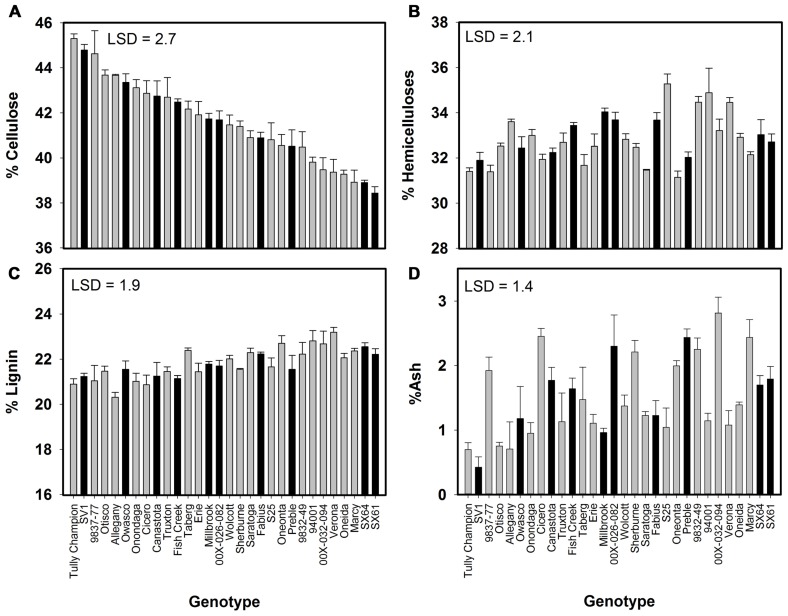
**Willow composition as percent dry weight (mean ± SE of three replicates) at harvest after 3 years of growth at Constableville, NY, USA as determined by high-resolution thermogravimetric analysis**. Black bars indicate 10 genotypes selected for pretreatment. **(A)** Cellulose, **(B)** hemicelluloses, **(C)** lignin, and **(D)** ash. Least significant differences (LSDs) from Tukey’s studentized range test are indicated on each graph.

**FIGURE 2:**
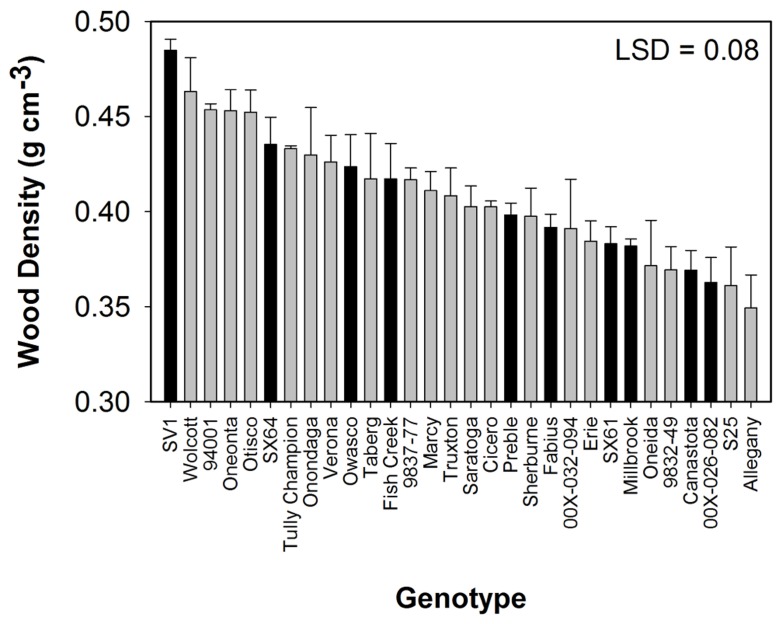
** Wood density (mean ± SE of three replicates) from 3 year growth at Constableville, NY, USA**. Black bars indicate 10 genotypes selected for pretreatment. Least significant difference (LSD) from Tukey’s studentized range test is indicated.

All compositional traits were significantly different (*P* < 0.05) by genotype (**Figure 1**). Cellulose showed the greatest variation among the genotypes ranging from 38 to 45%. ‘Tully Champion’ had the greatest cellulose content with low lignin, hemicelluloses, and ash content. The lowest lignin content was observed in ‘Allegany.’ The ash content was <3% of the total biomass in all cultivars. Wood density was significantly different by genotype with ‘SV1’ having the greatest density at 0.48 g cm^-3^ (**Figure 2**). Density ranged from 0.48 to 0.35 g cm^-3^.

### SUGAR RELEASE OF UNTREATED AND PRETREATED BIOMASS

**FIGURE 3:**
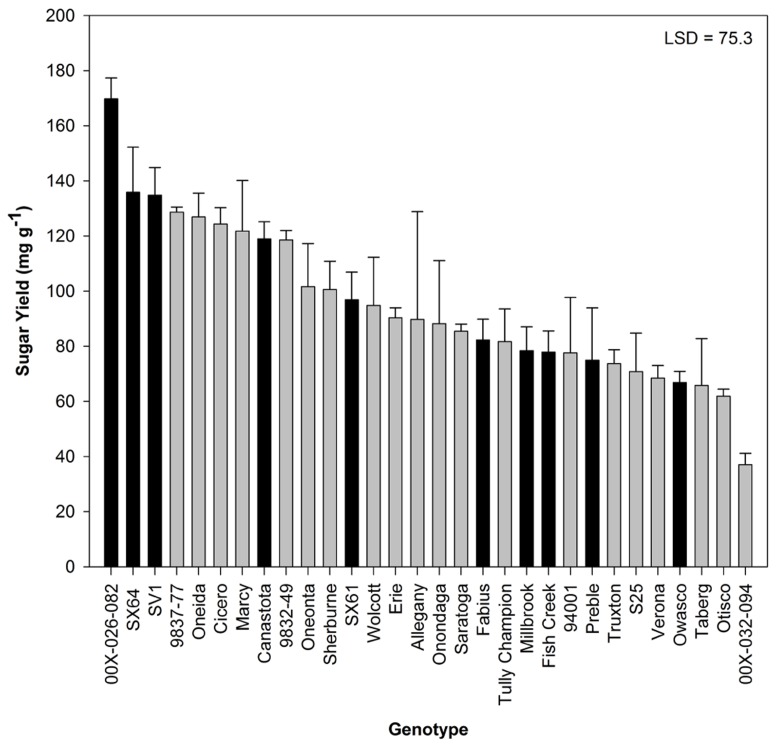
**Total sugar released (mean ± SE of three replicates) after enzymatic hydrolysis on untreated biomass**. Black bars indicate 10 genotypes selected for pretreatment. Least significant difference (LSD) from Tukey’s studentized range test is indicated.

**FIGURE 4:**
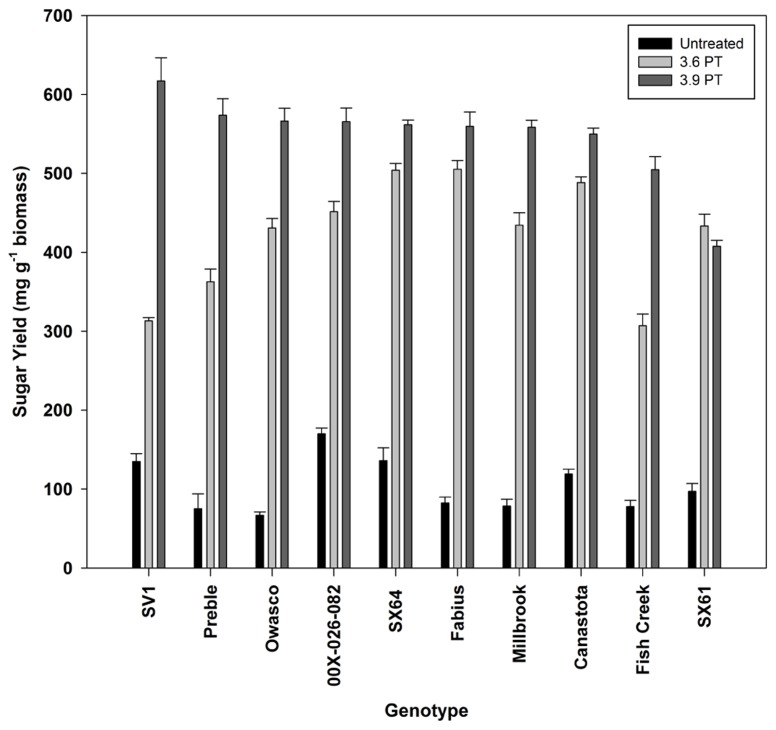
**Total sugar released (mean ± SE of three replicates) after enzymatic hydrolysis on untreated and hot-water pretreated biomass**.

Sugar release from untreated biomass was minimal, but there were significant differences by genotype (**Figure 3**). Based on this evaluation, 10 genotypes were selected for further analysis. Hot-water pretreatments improved sugar release in all experiments in a genotype-specific fashion (**Figure 4**). Except for ‘SX61,’ hot-water pretreatment with the 3.9 severity lead to greater sugar yields. The greatest sugar yield was observed in ‘SV1’ after the higher severity pretreatment of 3.9. After the 3.6 severity pretreatment, ‘Fabius’ had the greatest sugar released and only a small increase in sugar release was observed when the pretreatment severity was increased. For ‘SX61,’ there was no significant difference between the two pretreatment times.

### ETHANOL PRODUCTION BY SSF

**FIGURE 5:**
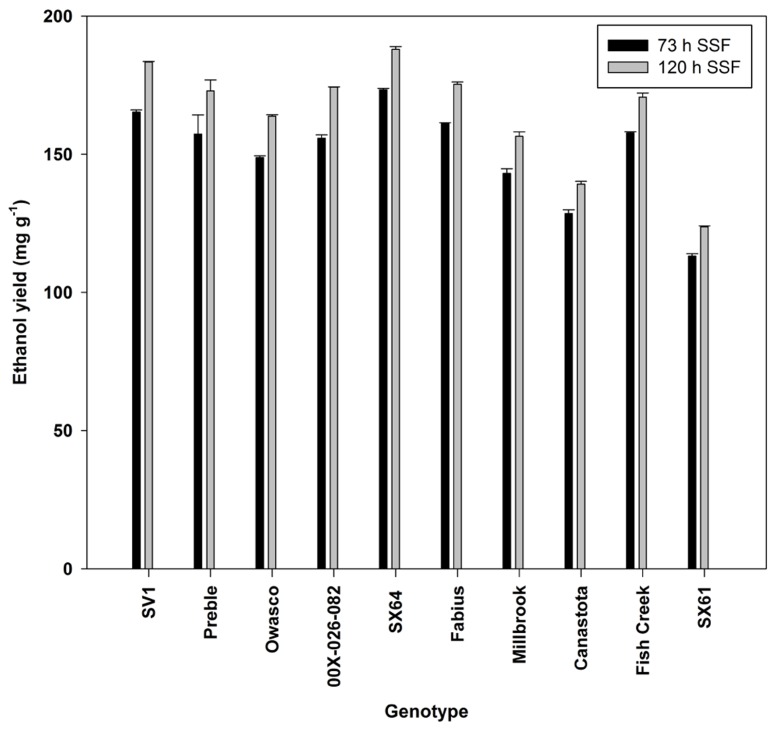
**Ethanol yield (mean ± SE of three replicates) from simultaneous saccharification and fermentation after two incubation times**.

Ethanol production was significantly different by genotype with the greatest ethanol yield from ‘SX64’ and ‘SV1’ (**Figure 5**). The increase in fermentation time from 73 to 120 h showed a significant increase in ethanol production across all genotypes, except for ‘Preble.’

### CORRELATIONS BETWEEN THE TRAITS

**FIGURE 6:**
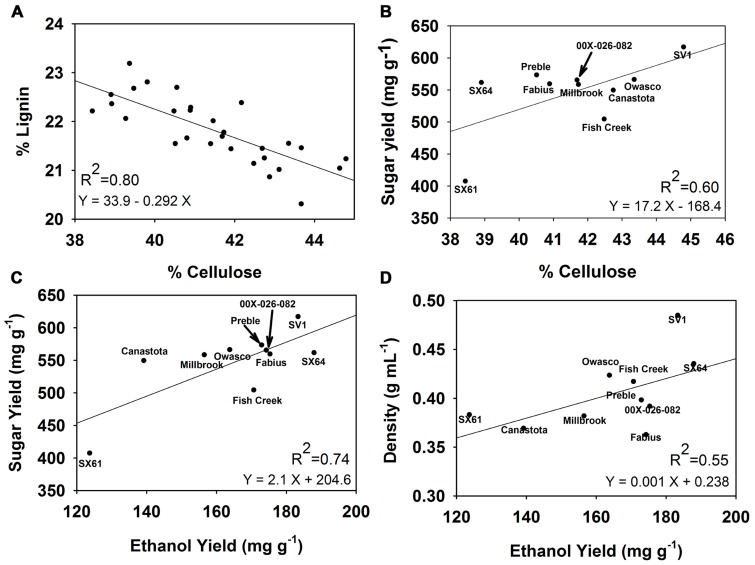
**Correlations observed among woody biomass traits, sugar release, and ethanol yield**. **(A)** % Lignin vs. % cellulose, **(B)** total sugar yield vs. % cellulose, **(C)** total sugar yield vs. ethanol yield, and **(D)** wood density vs. ethanol yield.

For the 30 genotypes studied in this trial there was a strong negative correlation between cellulose content and lignin content, with a correlation coefficient *R*^2^ = -0.80 (**Figure 6**). A weaker correlation was identified between cellulose content and sugar yields from the pretreatment with the higher severity with a correlation coefficient *R*^2^ = 0.60. Ethanol yields correlated positively with sugar yields (*R*^2^ = 0.74) and wood density (*R*^2^ = 0.55).

## DISCUSSION

To identify a relationship between biomass composition and sugar yield, biomass from shrub willow genotypes representing a range of compositional traits were pretreated using a hot-water method, and sugars were hydrolyzed enzymatically. In this study, enzymatic hydrolysis was performed with excess enzyme concentrations to reveal differences in biomass recalcitrance. Large differences in sugar yield were observed for the untreated biomass and for the two different pretreatment severities. These analyses were performed on bulk biomass samples including the bark, since commercial conversion facilities will utilize willow wood chips harvested with the bark. Bark is known to have a diversity of extractives and phenolic compounds present in greater levels than in debarked wood, many of which can act as fermentation inhibitors ( Jonsson et al., 1998; Robinson et al., 2002).

The range of compositional differences among the willow genotypes examined in this study was rather modest, 38.4–45.3% for cellulose, 31.1–34.9% for hemicelluloses, and 20.3–23.2% for lignin. In comparison, other recent studies have examined willow with cellulose content ranging from 34.8 to 41.8% and lignin ranging from 23.9 to 28.8% ( Ray et al., 2012) or lignin content ranging from 15.7 to 27.9% in *Populus trichocarpa* ( Studer et al., 2011). Selecting a larger and more diverse group of genotypes growing on more than one site may have increased the range in compositional variation used in this study. Although differences in recalcitrance and improved sugar release have been identified among natural variants ( Studer et al., 2011; Ray et al., 2012), those differences are more dramatic in plants engineered to have greatly reduced lignin content ( Chen and Dixon, 2007; Fu et al., 2011; Coleman et al., 2012; Mansfield et al., 2012; Pattathil et al., 2012). The extremes of lignin content have been extended through genetic engineering to contents as low as 10% in *Populus*, resulting in dramatic differences in enzymatic hydrolysis characteristics ( Mansfield et al., 2012). Within the range of compositional variation of non-genetically engineered willows, the differences in accessibility to hydrolytic enzymes may be due to more complex and subtle differences in lignocellulosic structures and polymer linkages.

Sugar yield from untreated biomass samples reached 170 mg g^-1^ biomass (17% of the biomass), indicating that there is available sugar in the willow biomass prior to pretreatment. The observed range of sugar yield (40–170 mg g^-1^ biomass) is comparable to other works with untreated poplar and willow ( Brereton et al., 2010; Studer et al., 2011). The majority of the sugar released was glucose (data not shown), but neither total sugar yield nor glucose yield was correlated with biomass composition or wood density, indicating that the total sugar content in the biomass is not the primary contributing factor for sugar availability in untreated biomass. The genotype 00X-026-082 had the highest sugar yield and only moderate levels of cellulose and hemicelluloses. The very low sugar yield observed in 00X-032-094 was due to undetectable levels of glucose. It was the only genotype to release no glucose monomers. It is unclear whether glucose was released and immediately degraded or if it never was a product of hydrolysis.

The hot-water pretreatment significantly improved the accessibility of the cell walls to enzymatic digestion, leading to three to five times greater sugar yields compared with untreated biomass. Sugar yields observed for willow in this study were comparable to those observed for poplar subjected to a similar pretreatment severity ( Studer et al., 2011). Sugar yield following the higher severity pretreatment was dependent on cellulose content in the biomass, based on the observed correlation (**Figure 6**), as was observed among European willow genotypes ( Brereton et al., 2010). This suggests that breeding for differences in biomass composition could have an impact on sugar availability for fermentation. ‘SV1’ which has the greatest cellulose content (44.8%) released the most sugar following the 3.9 severity pretreatment, 617 mg g^-1^ biomass, equivalent to 80% recovery. However, cellulose content alone cannot be the only factor impacting sugar release, since some of the genotypes with low cellulose content had high sugar yield, such as ‘Preble.’ Other factors influencing recalcitrance and sugar release include cell wall chemistry, such as phenolic groups in the lignin ( Lapierre et al., 2000), S:G ratios in the lignin ( Studer et al., 2011), and cell wall structural variability dependent on tissue types ( Dinus et al., 2001).

The differences in sugar release between the two different pretreatment severities provided insight into the recalcitrance of the willow genotypes. To reduce costs associated with biomass conversion, it would be highly beneficial to utilize feedstocks that have high sugar release following pretreatments with a lower severity. In this study, the less severe pretreatment of 10 min resulted in the greatest sugar yield from ‘SX64’ and ‘Fabius,’ while there was only a small increase in sugar release (from 70 to 80% recovery) when the biomass was exposed to the more severe pretreatment. These two genotypes were less recalcitrant than ‘SV1,’ which required the severe pretreatment to release most of the sugars.

Fermentation to ethanol produced 50% of the theoretical ethanol yield from ‘SX64’ and ‘SV1.’ Sassner et al. (2008) reported ethanol yields with willow from simultaneous SSF reaching 76% of the theoretical yield. A positive correlation with wood density was also identified. There is only limited research examining the relationship between wood density and sugar release or conversion to ethanol. A recent study examining sugar recovery following pretreatment and enzymatic hydrolysis from high and low density genotypes of 0.48 and 0.40 g cm^-3^, respectively, resulted in greater sugar recovery from the low density genotype ( Martin et al., 2011; Djioleu et al., 2012). However, only two genotypes were examined making it difficult to statistically identify a relationship between wood density and sugar release. Wood density is driven by fiber traits such as fiber lumen diameters, fiber wall to lumen ratios, and total wall areas ( Dinus, 2001; Martinez-Cabrera et al., 2009), so further studies considering the cell ultrastructure and wood anatomy of shrub willow are necessary to understand the relationships between wood density, sugar release, and ethanol yield.

Overall this study has shown significant variation in biomass composition, differences in sugar yield and recalcitrance to pretreatment, and differences in ethanol yield among the shrub willow genotypes. Relationships between sugar release and cellulose content were identified, as well as a relationship with ethanol yield. However, it is clear that an increase in cellulose content may increase sugar yield, but does not infer an increase in ethanol production. These findings will spur future research and large-scale evaluation of willow germplasm for variation in recalcitrance to promote future breeding efforts aimed at producing new willow cultivars with maximum bioconversion to ethanol without compromising biomass yield potential.

## Conflict of Interest Statement

At the time this research was performed, Haowen Xu and David A. Hogsett were employed by Macoma Corporation, a for-profit biofuels company. The other authors declare that the research was conducted in the absence of any commercial or financial relationships that could be construed as a potential conflict of interest.
